# Editorial: Novel approaches to overcome drug resistance in breast cancer

**DOI:** 10.3389/fonc.2026.1910442

**Published:** 2026-10-01

**Authors:** Arunima Biswas, Somsubhra Nath

**Affiliations:** 1 Molecular Cell Biology Laboratory, Department of Zoology, University of Kalyani, Kalyani, India; 2 Institute of Health Sciences, Presidency University, Kolkata, India

**Keywords:** chemotherapy, drug resistance, epithelial-to-mesenchymal transition (EMT), therapeutic effect, triple-negative breast cancer (TNBC)

Breast cancer recurrence, often driven by drug resistance, remains a formidable challenge in oncology. Despite major advances in traditional therapeutic strategies, advancement in personalized medicine, resistance across treatment modalities is a major hindrance which compromise long-term efficacy of the treatment and contribute to tumour progression, recurrence, and increased mortality. This Research Topic titled *‘Novel Approaches to Overcome Drug Resistance in Breast Cancer’* was conceived to examine the molecular, clinical, and translational dimensions of drug resistance in breast cancer and to highlight emerging strategies that may help overcome this persistent barrier. The collection brings together studies addressing mechanisms of resistance, biomarkers and diagnostics, clinical and translational investigations, evidence on treatment-resistant breast cancer, and technological innovations including AI, bioinformatics, advanced imaging, and genomic tools. By integrating laboratory discoveries with clinical application, this Topic aims to foster interdisciplinary collaboration and to advance more robust, sustainable, and patient-centred therapeutic strategies for breast cancer management.

The editorial aims to provide a concise but intellectually rich discussion devoted to one of the most persistent barriers in breast cancer management: therapeutic resistance. Significant advances have been achieved in the treatment of breast cancer with endocrine therapy, chemotherapy, targeted therapy and immunotherapy. But drug resistance remains a driver of recurrence, progression and mortality across breast cancer subtypes. The challenge is addressed in the collection published in the editorial by providing a range of studies that together argue for a broader and more integrated understanding of resistance. The editorial describes resistance as a multifactorial and dynamic phenomenon, not as a single molecular event. Resistance may be due to interactions of tumor environment, microenvironmental pressures, therapeutic selection, response assessment, and patient-related constraints that impact treatment delivery.

A major strength of the Research Topic, as pointed out in the editorial, is its conceptual breadth. The selected papers do not limit the discussion to one subtype of breast cancer, one treatment modality or one mechanism of resistance. Instead, they include cellular plasticity, subtype-specific therapeutic innovation, predictive biomarker development, experimental target discovery, optimization of treatment strategies, and supportive-care interventions to maintain therapeutic benefit. This breadth is reflective of the fact that resistance in breast cancer is not just a laboratory problem, but a translational and clinical one. In practice, treatment failure may occur due to molecular adaptation in tumor cells, incomplete response prediction, poor clinical endpoints, or an inability of patients to tolerate or complete therapy. The editorial pulls together papers on these various dimensions and therefore offers a contemporary perspective on resistance research as an interdisciplinary enterprise that demands coordination across molecular biology, pathology, imaging, clinical trial design, and supportive oncology.

Within this broader framework, one of the central discussions of the editorial is the review on epithelial-to-mesenchymal transition (EMT) and its role in breast cancer drug resistance. The importance of this contribution is that it attempts to re-position EMT as more than a process associated with invasion and metastasis. Rather, EMT is framed as a form of cellular plasticity that allows tumor cells to respond to therapeutic pressure and persist through treatment (1, 2). The editorial states that the review’s key contribution is “to systematically integrate EMT-associated signaling pathways, transcription factors and microRNA networks, thereby positioning resistance as a dynamic adaptive state rather than a fixed molecular defect.” This view is particularly relevant as it includes the increasing acceptance that resistant tumor cells do not simply acquire a single dominant mutation but rather may transition into altered phenotypic states that offer more extensive survival advantages under endocrine, chemotherapeutic or targeted therapy.

This EMT-centric review is considered a conceptual anchor for the collection as it addresses a process that covers a range of therapeutic contexts. And in doing so, emphasizes a critical paradigm shift in cancer biology: the move away from static models of resistance towards a more nuanced appreciation of hybrid states, lineage flexibility and context-dependent adaptation. At the same time, the editorial takes a measured stance on the review’s translational implications. EMT remains a heterogeneous and controversial concept with the relative importance of partial EMT states debated and the difficulty in translating preclinical mechanistic insights into clinically validated therapeutic strategies. The review is thus commended for providing a compelling biological framework and for probing the possibility that EMT-related pathways can be therapeutically targeted to resensitize tumors, with the ultimate value being more in clarifying the conceptual terrain than in providing immediately actionable clinical solutions.

Another dimension of the Research Topic is reflected in the review of therapeutic innovations in triple-negative breast cancer (TNBC), a subtype that remains one of the most clinically aggressive and treatment-refractory forms of breast cancer (3; Mir et al.; 4). We bring this article in this editorial as an important reminder that TNBC should no longer be viewed as a receptor-negative category defined by the absence of endocrine and HER2 targets. Rather it is now better conceptualised as a biologically heterogeneous disease with multiple candidate vulnerabilities. The review also covers a broad range of molecular and antibody-based therapeutics including targeted pathways, such as androgen receptor signaling, PI3K/AKT/mTOR, Notch, Wnt/β-catenin, Hedgehog, and TGF-β. This way it provides a broad map of the changing therapeutic landscape to the readers and demonstrates how TNBC research is moving beyond the historical dependence on cytotoxic chemotherapy alone.

The Research Topic highlights particularly the translational relevance of combining molecular targeting with monoclonal antibody-based strategies. This convergence reflects an emerging trend in the management of TNBC, where selective pathway inhibition, immune engagement, and microenvironmental modulation are increasingly being considered in concert, rather than in isolation. Thus, the strength of the article is its presentation of TNBC as a changing therapeutic landscape with a growing armamentarium of biologically based interventions. But the editorial also notes an important caveat: the clinical readiness of these strategies varies widely. Some are already supported by meaningful translational data, while others are still exploratory. So, the review is most successful as a broad overview of therapeutic diversification rather than a definitive guide to resistance management for standard of care. Nonetheless, it provides a valuable addition to the collection as it places drug resistance in the context of the rapidly evolving landscape of TNBC therapy.

Among the original research articles, the work on circWWC3 and pathological complete response (pCR) after neoadjuvant therapy in TNBC stands out as one of the most directly translational contributions. Its significance is the integration of molecular biomarker assessment with immune and imaging factors to develop a predictive nomogram for treatment response. In particular, the study integrates circWWC3 expression, tumor-infiltrating lymphocytes, and diffusion parameters obtained from MRI, thus demonstrating a multimodal approach for response prediction. Given the inability of any single biomarker in isolation to adequately address the complexity of therapeutic sensitivity in TNBC, the editorial is correct to see this as an important avenue for breast cancer research. The study goes beyond purely computational association by integrating tissue-based, radiologic, and clinicopathologic features from a defined patient cohort towards a more clinically relevant model of stratification.

The real notability of this study lies in its layered approach. By blending molecular clues with imaging data, it paints a far richer picture of how triple-negative breast cancer responds to treatment than any single factor ever could. The discovery that high levels of circWWC3 might predict which patients will respond well to therapy is particularly exciting, hinting at a future where we could tailor treatments more precisely (5). That said, the authors wisely pump the brakes: these findings are still early, and we’ll need rigorous testing across different patient groups before this becomes a go-to tool in the clinic. For now, circWWC3 looks more like a promising signpost than a proven driver of treatment success. Nevertheless, this study marks a meaningful shift, moving us beyond just identifying biomarkers toward modelling how patients respond, which is exactly what we need to crack tough, resistant cancers.

The significance of this question cannot be overstated, for it reveals a critical yet often overlooked dimension of therapeutic resistance: its dependence not merely on the molecular escapism of cancer cells but also on the very frameworks we employ to measure success. What if the tools we use to assess therapeutic efficacy are themselves misaligned with the biological realities of disease subtypes? In such cases, even the most promising strategies risk being dismissed prematurely, their potential obscured by endpoints that fail to capture their true value. The editorial astutely highlights this tension, noting that the endocrine-based regimen in question not only demonstrates improved tolerability (a factor that amplifies its clinical relevance) but also exposes the fragility of our evaluative paradigms. Yet, the study’s limitations are impossible to ignore. Its observational design, reliance on propensity score matching, and the modest size of the cohort receiving endocrine therapy combined with dalpiciclib introduce layers of uncertainty. These constraints compel a cautious interpretation, framing the findings as hypothesis-generating rather than conclusive. Nevertheless, the paper’s true merit lies in its call for a more sophisticated approach to treatment design and response assessment in luminal breast cancer, one that acknowledges the intricate interplay between biology and methodology in unravelling the enigma of therapeutic resistance.

At a more foundational level, the editorial turns its attention to the study of ESCO2 inhibition in breast cancer, positioning it as a compelling exemplar of mechanistic target discovery (6). Here, the focus shifts to a relatively uncharted vulnerability, where ESCO2 knockdown is shown to trigger cell-cycle arrest and apoptosis through a cascade of tightly regulated pathways, including the p53-CDK1 axis and the BAX/Bcl2/caspase signaling nexus. What sets this work apart is its reliance on functional perturbation rather than mere correlative associations, a distinction that lends it a rare mechanistic credibility. Such investigations are indispensable to the broader endeavour of overcoming resistance, for they illuminate previously underappreciated biological dependencies that may serve as the bedrock for future therapeutic innovations.

Yet, the editorial carefully draws a distinction between this study and the more translationally oriented contributions in the collection. While its contributions to tumor biology and target identification are undeniable, its implications for surmounting established drug resistance remain, for now, indirect. The path from mechanistic insight to clinical application is fraught with challenges, and the study’s findings, though provocative, are but a preliminary step. To bridge this gap, further exploration in resistant models and clinically relevant systems will be essential to determine whether ESCO2-related pathways can be harnessed therapeutically. Even so, the study’s inclusion in the Research Topic serves a broader purpose: it underscores the necessity of looking beyond incremental refinements to existing therapies and instead embracing the discovery of entirely novel biological intervention points.

A particularly striking feature of the editorial is its inclusion of the Home-Combo study protocol, which investigates a supervised, online, home-based exercise intervention for women undergoing neoadjuvant chemotherapy. This paper is notable not only for its innovative approach but also for its expansion of the very concept of resistance. In the complex landscape of real-world oncology, poor outcomes are not solely the result of cancer cells outmanoeuvring therapy; they are equally a consequence of treatment regimens compromised by toxicity, interruptions, delays, or outright discontinuation. By improving treatment completion rates or preserving functional capacity, interventions like Home-Combo may indirectly yet meaningfully enhance oncologic outcomes. The protocol’s selection of chemotherapy completion rate and tumor regression as primary endpoints represents a conceptual leap: it reframes supportive care not as a mere adjunct to quality of life but as an integral component of treatment efficacy.

Moreover, the protocol’s online and home-based design amplifies its practical relevance, addressing the pressing concerns of scalability and accessibility that dominate contemporary oncology care. In this regard, the study introduces a patient-centred, implementation-focused perspective that complements the molecular and biomarker-driven narratives elsewhere in the collection. However, as a protocol, its contributions remain prospective, its ultimate significance contingent on the trial’s results, adherence to the intervention, and the interpretability of any observed links between exercise and treatment outcomes. Despite this uncertainty, its inclusion is justified, for it broadens the field’s understanding of resistance to encompass not only the molecular adaptations of tumors but also the conditions that enable patients to fully engage with and benefit from therapy.

In a nutshell, the articles in this Research Topic address fundamental mechanisms of resistance, predictive biomarker development, therapeutic innovation, mechanistic target discovery and supportive-care interventions. Overall, the Topic illustrates that overcoming breast cancer drug resistance requires an integrated translational approach that links molecular biology, imaging, clinical decision-making and patient-centred care.

In conclusion, it can be inferred that the collected papers do not offer a single explanation or solution, rather, they collectively show that overcoming resistance requires a synthesis of mechanistic insight, translational precision, methodological rigor, and patient-centred care. Future progress will depend on validating biomarkers in larger cohorts, testing candidate targets in resistant models, refining response assessment across disease subtypes, and integrating clinical, imaging, and computational tools into more robust decision-making frameworks. Supportive strategies that improve treatment adherence and tolerability should be recognized as part of resistance management rather than as secondary considerations. In this light, the editorial transcends its role as a summary of individual studies and instead presents a forward-looking framework for research aimed at achieving more durable and meaningful improvements in breast cancer outcomes.
